# Morphological and Molecular Variability of *Alternaria solani* and *Phytophthora infestans* Causing Tomato Blights

**DOI:** 10.1155/2023/8951351

**Published:** 2023-05-27

**Authors:** Lydia Mugao

**Affiliations:** Department of Agricultural Resource Management, University of Embu, P.O. Box 6-60100, Embu, Kenya

## Abstract

*Alternaria solani* and *Phytophthora infestans* cause early and late blight diseases in tomato and potato, respectively. *A. solani* can survive for more than a decade in the soil, seed, or in plant residues at optimum temperature. The pathogen exhibits high molecular and genetic variation between isolates from potato and tomato plants, in different countries. Morphological studies reveal separate conidia borne singly on simple conidiophores. Spores are elongated, muriform, beaked, septate, and dark coloured. The mycelia are branched and septate. *A. solani* demonstrated a high genetic variability among isolates originating from the United States, Greece, Cuba, Canada, Russia, Turkey, South Africa, Brazil, and China based on vegetative compatibility groups and molecular markers (random amplified polymorphic DNA markers, random amplified microsatellite markers, and amplified fragment length polymorphisms). Different morphological and molecular variations indicate the presence of variability among the isolates. On the other hand, *P. infestans* is a diploid, obligate, heterothallic, and biotrophic oomycete, whose asexual lifecycle is characterized by alternating phases of sporangia germination, hyphal growth, and sporulation. The mycelia of *P. infestans* is coenocytic, multinucleate, and aseptate although the cross walls do not form in old cultures. Sporangia are borne singly on the branch tips of the alternately branched sporangiophore. Sporangium is hyaline and lemon shaped with a papilla at the distal end. Mating types A1 and A2 with different clonal lineages have been discovered in various parts of the world indicating variation in the species.

## 1. Introduction

*Alternaria solani* causes early blight disease in tomato. The pathogen belongs to the phylum Ascomycota, class Deutromycetes, and order Moniliales [1]. *A. solani* is a soil inhabiting fungus and spreads to host plants through air and rain splash [2]. The pathogen is among the large-spored group of the genus *Alternaria* which is distinguished by separate conidia borne singly on simple conidiophores. Its spores are elongated, muriform, beaked, septate, and dark coloured, while the mycelia are branched and septate [3]. The fungus survives as parasitic and as a saprophytic organism [4]. Early blight disease was first identified on potato, and the causative pathogen was named *Macrosporium solani and* was later classified under the genus *Alternaria* based on the spore development in chains in a culture medium [5]. The pathogen can survive for more than ten years in the soil, seed, or in plant debris at optimum temperature [6]. The fungus produces toxins such as alternaric acid, altersolanol, macrosporin, and zinniol that act on the protoplasm of the host and distract plant defense mechanisms [2].

*A. solani* exhibits a high genetic variation between isolates from potato and tomato plants and from different countries due to pathogen specialization and pesticide resistance [7]. *A. solani* fungal isolates from different host plant species vary in aggressiveness, physiology, and genetic diversity when inoculated in different plants [7]. When environmental conditions are favourable, the conidia germinate and form one or more germ tubes which penetrate the epidermal cell of the host through the stomata or directly by means of appressoria or wounds by hyphal growth [2]. The pathogen colonizes the host by degrading the cell walls of the host by producing toxins that destroy the cells of the host, therefore enabling them to acquire nutrients from the plant cells [8]. The toxins such as alternaric acid, altersolanol, macrosporin, and zinniol act on the protoplasm of the host and disrupt physiological processes that sustain the health of the plants [2]. The pathogen can survive for more than a decade in the soil, seed, or in plant residues at optimum temperature [9]. The pathogen causes early blight disease on tomato plant foliage. The disease infects all above ground parts of the tomato plant at all stages of the plant growth and development [10]. Early blight disease can cause 79% crop loss in tomatoes [11]. Quantity losses to early blight disease on tomato amount to 2.15% in resistant varieties and 42.75% in susceptible varieties [12].

*Phytophthora infestans* causes late blight disease in tomatoes and other solanaceous plants [13]. The genus name *Phytophthora* is coined from two Greek words; “Phyto” meaning plant and “phthora” meaning destroyer [14]. The pathogen results in massive destruction on tomatoes [15]. *P. infestans* belongs to the kingdom: Chromista, phylum: Heterokontophyta, class: Oomycota, order: Peronosporales, and family: Pythiaceae [16]. *P. infestans* is a diploid, obligate, heterothallic, and biotrophic oomycete, whose asexual lifecycle is characterized by alternating phases of sporangia germination, hyphal growth, and sporulation [17]. Morphologically, the pathogen is similar to fungi but phylogenetically related to brown algae or diatoms because their sexual oogonia and antheridia resemble those of brown algae. The pathogen is sometimes referred to as water mould because of its special adaptations to water habitat [18]. The pathogen's cell wall is made of cellulose and other glucans unlike fungi whose cell walls are made of chitin [19]. The genus *Phytophthora* has no ability to synthesize thiamine and sterol because it lacks the synthesis pathways and therefore acquires these essential compounds from the host plant [17].

*P. infestans* is a diploid, obligate, biotrophic, and heterothallic pathogen with two mating types: A1 and A2 [20]. The pathogen is an oomycete, and its asexual lifecycle is characterized by alternating phases of hyphal growth, sporangia germination, and sporulation [21]. Sexual reproduction results to oospores that are thick walled to enable them overcome harsh environmental conditions such as cold, chemical fumigations, and microbial degradation, thus conserving the inocula for the subsequent years [22]. The pathogen has low levels of diversity, and its population structure and distribution are influenced by host preference [23]. The mycelia of *Phytophthora* is coenocytic, multinucleate, and aseptate although the cross walls do not form in old cultures [24]. Sporangia are borne singly on the branch tips of the alternately branched sporangiophore [25], while the sporangium is hyaline and lemon shaped with a papilla at the distal end [26].

Sporangia are released from the sporangiophores by twisting and popping of the sporangiophore, and they are disseminated by air currents [27]. The zoospore formation within the sporangia is temperature dependent. Vegetative spores and mycelia can exist and remain infectious in plant debris for not less than one week and continue to cause infections when they are in contact with susceptible plant tissues [25]. The pathogen can grow on selective culture media such as rye agar, lima bean agar, pea agar, corn seed agar, and V-8 agar [19]. In the culture, *P. infestans* grows slowly and the mycelia are white and fluffy although some isolates have lumpy appearance [17].

The sexual cycle of *P. infestans* occurs when mating types A1 and A2 meet and release hormones that trigger the formation of oospores [15]. For the production of oospores, both the A1 and A2 mating types are needed. However, the presence of the two types is not a guarantee of sexual reproduction since some levels of incompatibility exists [28]. The mating types are compatibility types, differentiated by mating hormones, but are not dimorphic forms. When the mycelia of type A1 and A2 mating types interact, the mating hormones induce gametangial formation in the opposing mating types, initiating sexual propagation by means of oospore formation [29]. The mycelia undergo meiosis during gametangia formation to form antheridia and oogonia that are haploid. The antheridium fuses with an oogonium during the sexual life cycle to form a diploid oospore [30]. Sexual reproduction results to gene recombination that generates new virulent strains, thus complicating the management of the disease [31].

## 2. Morphological Diversity of *Alternaria solani*

### 2.1. Colony Radial Growth

Studies have reported that the colony growth varies between *A. solani* races subjected to the same growth conditions. According to Kumar et al. [32], the radial growth ranged from 14.9 mm to 57.7 mm among eleven isolates investigated. Few isolates grew fast within the first four days, others increased growth in days 6-7, while another lot increased its growth on the last days of the experimental period [32]. Nikam et al. [33] investigated the radial growth of eight *A. solani* isolates and reported radial growth ranging between 65.80 mm and 88.50 mm. Similarly, Nafisa and Javed [34] evaluated seven isolates of *A. solani* and reported their growth to range between 50 mm and 88 mm within a period of seven days. One of the isolates had the slowest growth and attained a radial growth of 50 mm by the end of the experimental period. One of the isolates grew to 60 mm, two with 65 mm, one with 79 mm, and two with 88 mm. Differences in radial growth among isolates signify variability. The radial growth obtained from two isolates studied by Rahmatzai et al. [35] was 79 mm for isolate one and 61 mm for isolate two.

### 2.2. Colony Growth Pattern

The fungal colony growth pattern also varies between isolates which is an indication that there are many strains. For example, according to Kumar et al. [32], some of the isolates had circular or irregular growth margins, while some had rough and others with smooth margins. Among the 19 fungal isolates studied by Mugao et al. [36], majority of them (12) had a growth margin that was irregular, while the others (7) had a circular growth margin. Among eight isolates studied by Nikam et al. [33], the growth margins were either circular or regular and either smooth or rough. Some had zonation, while others were without zonation. Results of Nafisa and Javed [34] also showed that the growth margins of the seven isolates studied were regular and smooth and there was none with an irregular growth margin. Loganathan et al. [37] studied growth patterns of 5 *A. solani* isolated and reported that 3 had a regular growth pattern, while 2 had an irregular growth pattern.

### 2.3. Colony Colour/Pigmentation

The colour of *A. solani* varies among races. According to Kumar et al. [32], some of the *A. solani* isolates appeared black, others had yellow pigmentation and appeared greenish, while others were brownish black. Similar results were reported by Mugao et al. [36], and after evaluating nineteen isolates, the colony colour varied from being greenish brown, dark brown, grey, to being greyish brown. Nikam et al. [33] evaluated the morphology of eight *A. solani* isolates, and only two out of the eight isolates had a similar colony colour which was brownish black. The other six isolates had different colony colours (black, dark grayish, olivaceous black, pinkish red, light grey, and creamish white). According to Nafisa and Javed [34], the seven isolates studied had different colony colours. Two of them were blackish brown, three were dark brown, and two were black in colour. Studies of Loganathan et al. [37] also revealed variations in *A. solani* colony colour. Out of the 5 isolates studied, four of them had grey colony colour, while one was yellowish grey. Upadhyay et al. [38] revealed a high degree of variation of colony colour of 32 isolates studied. Among them, two were black, 3 were grey, 2 were grey with white pigmentation, and 3 were grey with cream pigmentation among many other colour combinations. Results of Rahmatzai et al. [35] revealed reddish black pigmentation and brownish black pigmentation on the two isolates studied, while black with pale red pigmentation and dark grey to black with dark pigments were shown on two isolates studied by El-Ganainy et al. [39].

### 2.4. Conidial Morphology

#### 2.4.1. Conidial Length and Thickness

Research has shown that conidial length and thickness of *A. solani* varied between different isolates, indicating the presence of different races. Reports of Kumar et al. [32] showed that the length of the conidia also varied ranging from 150 to 300 *μ*m, while thickness ranged between 15 and 19 *μ*m. Ahmad [40] studied the conidial morphology of *A. solani* and discovered that some have large conidia of 175 *μ*m. Studies conducted by Nafisa and Javed [34] revealed conidial width ranging between 10 and45 *μ*m. According to Loganathan et al. [37], the conidial length of the five isolates studied ranged between 80 and 97 *μ*m and the thickness ranged between 23 and 32 *μ*m. Two of the *A. solani* isolates studied by Rahmatzai et al. [35] showed varied conidial length and width. The first isolate had a higher conidial length of 25–44 *μ*m and a width of 7–15 *μ*m, while the second one had 20–30 *μ*m length and 5–13 *μ*m width. The isolates studied by Naik et al. [41] showed 19.5–70 *μ*m conidial length and 6–22 *μ*m width. Other isolates had conidial length ranging from 70.63 to 77.61 *μ*m and a width of 16.94–25.05 *μ*m [39].

#### 2.4.2. Conidial Colour

Research has shown that the colour of the fungal conidia varied with isolates indicating variability. For example, according to Kumar et al. [32], the conidia colour varied from dark muriform, pale golden, to olivaceous brown. These results were in consonance with those reported by Nafisa and Javed [34]. However, Rotem [8] reported that the conidia of *A. solani* are brown, grey, or dark coloured with high content of melanin that protects them against diverse environmental conditions including resistance to microbes and hydrolytic enzymes.

#### 2.4.3. Conidial Beak Size and Shape

Studies on *A. solani* have revealed that their beak sizes and shapes vary within races. According to Ahmad [40], beak length ranged between 47 and 65 *μ*m. Some have their beaks branched, while others have unbranched beaks [32]. Mugao et al. [36] evaluated the beaks of 19 isolates where twelve isolates had elongated and unbranched beaks. Six of the isolates had slender, short, unbranched beaks, while one had an elongated branched beak. The report of Nikam et al. [33] also showed that the beak sizes also varied with some having elongated beaks, while others had short beaks. Seven strains of *A. solani* studied by Nafisa and Javed [34] revealed a beak size ranging between 10 and 50 *μ*m. According to Loganathan et al. [37], the beak length of the 5 isolates studied ranged between 60 and 89.3 *μ*m. Rotem [8] reported that the length of the beak size of *A. solani* conidia ranged between 139 and144 *μ*m and the beak width ranged between 12–20 *μ*m. According to Simmsons [42], the conidial sizes were 120–296 *μ*m length and 12 and 20 *μ*m width. Rahmatzai et al. [35] reported that the two isolates studied had a varying beak size of 6–10 *μ*m and 4–8 *μ*m, respectively, while El-Ganainy et al. [39] had isolates with beak length ranging between 27.91 and 116.61 *μ*m.

#### 2.4.4. Conidial Septation

*A. solani* isolates evaluated by different researchers revealed that the conidia were septate, but the number of septa varied between isolates. For example, according to Kumar et al. [32], the number of conidia septa ranged between 9 and 11 transverse septa and 1–4 longitudinal septa. Mugao et al. [36] studied the conidia septa of 19 *A. solani* isolates and concluded that the number of conidia septa also varied between the isolates with thirteen of the isolates having four transverse septa but no longitudinal septa. The study further revealed that three of the isolates had three transverse septa and out of the three, one of the isolates had one-longitudinal septa, but the other two did not have. Nikam et al. [33] reported that the conidial septation varied between the isolates with the transverse septa ranging between 5 and 12, while the longitudinal septa ranged between 1 and 4. Seven isolates of *A. solani* studied by Nafisa and Javed [34] also showed septa variation. The longitudinal septa ranged between 3 and 10, while the transverse septa ranged between 1 and 3. However, results of Simmsons [42] and Singh [4] showed that the conidia contained 5–10 transverse septa and 1–5 longitudinal septa. Rahmatzai et al. [35] reported varying conidial septation from the two isolates studied. The first isolate had 2–11 transverse septa and 1–3 longitudinal septa, while isolate two had 2–8 transverse septa and 1–3 longitudinal septa. According to Naik et al. [41], the transverse septa of *A. solani* studied ranged between 2 and 7, while the longitudinal septa ranged between 1 and 4. However, two isolates studied by El-Ganainy et al. [39] had more longitudinal septa (5–11 and 2–5) than the transverse septa (3–7 and 0–3).

### 2.5. Mycelial Morphology

According to Nikam et al. [33], the eight isolates of *A. solani* studied had different mycelial widths (6.42 *μ*m, 6.20 *μ*m, 4.50 *μ*m, 4.26 *μ*m, 3.73 *μ*m, 3.64 *μ*m, 3.50 *μ*m, and 2.54 *μ*m), indicating variability among the isolates. Nafisa and Javed [34] evaluated mycelia of seven isolates and reported smaller mycelial width ranging between 0.9 *μ*m and 1.6 *μ*m and were septate. Reports of Rotem [8] revealed that the mycelia of the studied isolates were septate, branched, and melanised. They were either grey, black, or olive in colour. Similar results were reported by Simmsons [42], but the colour was light brown that turned darker with age. The mycelial size of two isolates studied by Rahmatzai et al. [35] was 25 *μ*m and 2 *μ*m, respectively.

## 3. Molecular Diversity of *Alternaria solani*

### 3.1. PCR Amplifications

*Alternaria solani* pathogen exhibits a high genetic variation between isolates from tomato and potato plants in different countries [32]. *A. solani* demonstrated a high genetic variability among isolates originating from the United States, Greece, Cuba, Canada, Russia, Turkey, South Africa, Brazil, and China based on vegetative compatibility groups [43] and molecular markers (randomly amplified polymorphic DNA markers, randomly amplified microsatellite markers, and amplified fragment length polymorphisms) as reported by Pérez Martínez et al. [44]. Nikam et al. [33] conducted a molecular study of 8 isolates using RAPD-PCR analysis, and the amplification process regrouped them into two groups, and each group had an 80% similarity coefficient. PCR products obtained by Nafisa and Javed [34] on seven strains revealed variations in band sizes ranging between 518 and 549 bp using ITS primers. Previously, *A. solani* species yielded band sizes ranging between 400 and 600 pb using ITS primers [45, 46]. Reports of Loganathan et al. [37] showed that the ITS regions of five isolated amplified produced a similar band size of 580 bp. According to Upadhyay et al. [38], 32 isolates of *A. solani* DNA amplified using different primers yielded band sizes ranging between 200 and 1500 bp, an indication of variability among isolates.

### 3.2. Gene Sequencing to Study Genetic Diversity of *A. solani*

Different countries have used different methods to study genetic diversity of *A. solani*. For example, Naik et al. [41] studied genetic diversity among four *A. solani* isolates and reported that three of the isolates had 73.78% similarity, while the other one shared 45% genetic similarity, indicating distinct polymorphism. In Europe, *A. solani* carries two types of mitochondrial DNA referred to as genotype 1 (GI) and genotype 2 (GII) [47]. Seven isolates studied by Nafisa and Javed [34] were reported to have different accession numbers (MF539619, LC339936, EU315064.1, and JF491202.1) from GenBank, but they were all *A. solani* of different strains with 97–100% similarity with those in GenBank. Reports of Mugao et al. [36] revealed that all the 19 isolates were *A. solani* with different accession numbers (LN879928, MN871610, MN871616, and MN871613) indicating the differences in the genetic makeup. According to Loganathan et al. [37], the five isolates of *A. solani* studied had the same genetic makeup with all of them possessing the same accession number (JF796068) from GenBank.

Studies have shown that *A. solani* from the same lesions can be genetically distinct, indicating the high variability of the pathogen [32]. These genetic variations may be brought about by mutations, selection, and gene flow, and this influences disease dynamics and fungicide resistance [44]. Reports of Leiminger et al. [48] showed that twenty isolates obtained from identical geographical sites formed different subgroups, having distant similarities that ranged between 75 and 95%. Van der Waals et al. [43] studied 46 isolates of *A. solani* and reported that 44 of them were unique genotypes and the gene diversity was 0.27. Taylor et al. [49] revealed a high genetic diversity of *A. solani* using VC and RAMS markers. McDonald and Linde [50] also reported high genetic diversity among *A. solani* isolates and concluded that it poses a high risk of breaking down genetic resistance in the host plants. Isolates identified as *A. solani* from potato and tomato host plants showed large genetic distances based on RAPD analysis in the United States [51] and by AFLP markers in Cuba [44]. Tiwari and Chitora [52] isolated DNA from 12 isolates, and gene sequencing alienated the isolates into three cluster groups. Group one contained 10 isolates, while groups two and three were made up of one isolate each. Similarly, reports of Upadhyay et al. [38] revealed that the gene sequence of 32 isolates of *A. solani* grouped them into two groups, but group one had two subgroups depending on similarity percentage from GenBank. In USA, high genetic diversity of isolates from tomato and potato plants was detected using isozymes [53].

## 4. Morphological and Molecular Diversity of *Phytophthora infestans*

### 4.1. Morphological Diversity of *P. infestans*

*P. infestans* has white fluffy mycelia with beige tones caused by the presence of sporangia with hyphae measuring 5–8 *μ*m in diameter, hyaline and ramified. The mycelia were white in colour and aseptate with long sporangiophores, 5 *μ*m pedicel, and ellipsoidal sporangia [54]. Reports of Dida et al. [55] showed a total of 8 shapes of sporangia in Cameroon. These shapes were as follows: oval to ellipsoid, ellipsoid, pip form, elliptic, subglobose, globose, ovoid, and lemoniform, implying diversity among them. The sporangia length ranged between 0.63 *μ*m and 0.96 *μ*m, while the width ranged between 0.36 *μ*m and 0.50 *μ*m, respectively. Studies carried by [56] in Ethiopia revealed two distinct lineages. The Ia mtDNA haplotype lineage had the sporangia that were ellipsoid to ovoid, caduceus, and semipapillate. According to Shimelash and Dessie [56], the sizes of sporangia from the studied *P. infestans* averaged 60.5 *μ*m length and 31.7 *μ*m width. The sporangia were mostly ellipsoid to ovoid. They were caduceus and semipapillate for Ia mtDNA haplotype lineages of *P. infestans* and papillate for Ic mtDNA haplotype lineages of *P. infestans* [56]. This lineage also revealed the presence of chlamydospores, but all of them were of A1 mating type. The Ic mtDNA haplotype lineage had sporangia that were ellipsoid to ovoid, caduceus, and papillate arranged in simple and sympodia sporangiophores. The sizes of sporangia averaged 60.5 *μ*m in length and 31.7 *μ*m in width. The Ic mtDNA haplotype lineages had chlamydospores at the terminal hyphae. The two haplotype lineages were of A1 mating type.

### 4.2. Genetic Diversity of *Phytophthora infestans*

Mitochondria and the DNA in the nucleus of *P. infestans* have been used to evaluate its population structure and evolutionary history, and there are two mating types that coexist and can reproduce and survive sexually [25]. Although *P. infestans* undergoes frequent genetic changes [57], the pathogen portrays low genetic diversity and its distribution and population structure is influenced by the host preference [58]. Each host group of *P. infestans* is associated to different clonal lineage: tomato with US-1, potato with EC-1, wild solanaceous species with EC-2, and *S. betaceum* EC-3 [58]. *P. infestans* has been classified into four main groups based on mitochondrial haplotypes: Ia, Ib, IIa, IIb [31], and 1c [59]. The EC-2 and EC-3 lineages has been associated with the Ia haplotype, the US-1 lineage to the Ib haplotype, the US-6 to the IIb haplotype, the EC-1 to the IIa haplotype [31], and EC-2 has also been related to haplotype IC and correlated with new species, *P. andina* [59].

Hussain et al. [60] identified two *P. infestans* mating types A1 and A2, and all had amplicons of 524 bp using SCAR markers. Reports of Arafa et al. [61] identified *P. infestans* mating type A1 using SSR markers in Egypt with seven genotypes among fourty isolates indicating genetic variability. According to Santana et al. [62], 131 potato and 32 tomato isolates were evaluated where 59 isolates were classified as A1 mating type, while 103 were A2. All the tomato isolates that were of A1 mating type presented 86/100 pattern for the enzyme GPI and mtDNA Ib, indicating that the isolates belonged to the US-1 clonal lineage. Of the 131 potato isolates, 103 were A2, while 27 were of A1 mating type. Among the potato isolates, 27 exhibited the Gpi phenotype 100/100, the same as BR-1 (mating type A2) and 20 were 86/100, the same as US-1 (Mating type A1). Studies conducted in Brazil revealed the presence of the two mating types of *P. infestans* [63]. The US-1 lineage (mating type A1) is said to have many variants with at least 19 US genotypes [64].

In Russia, a study carried out by Beketova et al. [65] reported more (70%) A2 mating type than (30%) A1 in the study area. According to Cooke et al. [66], a clonal lineage of a new population known as 13_A2 which is also known as Blue-13 was discovered and was said to be more aggressive in Switzerland and Denmark. In UK and France clone, 6_A1 was discovered [66]. In Ireland genotype, 8_A1 of *P. infestans* was also discovered [67]. The population of *P. infestans* in Ethiopia had been described in two studies where the majority of all the isolates were described as Ia mitochondrial DNA haplotype with A1 mating type [68]. In Canada and the United States, two major genotypes of the pathogen, an A2 mating type (US-8) and an A1 mating type (US-11), were discovered [69, 70]. The presence of US-14 genotype of *P. infestans* and another isolate with a unique finger print were identified in New Jersey indicating variability [70]. *P. infestans* population of A2 mating type (US-22) was also identified in the United States and Canada [71]. Furthermore, clonal lineages US-23 and US-24 have also been identified in the United States with some important differences within the lineages [72].

Vargas et al. [73] conducted a study in Peru and Ecuador and found out that among the 64 isolates 59 were mating type A1, while the rest were mating type A2. PCR amplification performed by Trout et al. [74] on *P. infestans* collected from Tennessee, north and south Carolina, using PINF and ITS5 primers revealed band sizes of 600 bp. Sixty-five samples collected from Jordan valley and analysed by Wesam et al. [75] through PCR amplification yielded 990 pb. Khalid et al. [76] amplified the genomic DNA of *P. infestans* using ITS 3 and ITS 4 primers and yielded a band of 612 bp, while amplification with PINF and ITS 5 primers yielded a band of 600 pb [74]. Variations in band sizes indicate variability in their DNA.

## 5. Conclusion

The knowledge of genetic diversity of plant pathogens is important in selection of resistance genes in breeding programs. Therefore, the use of decision support systems should be incorporated to the management of early blight and late blight on potato and tomato crops to optimize fungicide spraying, avoiding environmental contamination, and the selection of resistant isolates. Preventive control with protectant fungicides must be always conducted. Further studies are necessary to reveal the aggressiveness, distribution, symptoms' pattern in the field, and difference in fitness of the species.
